# Immersive virtual reality or computerised mindfulness meditation for improving mood? Preliminary efficacy from a pilot randomised trial

**DOI:** 10.3389/fpsyg.2023.1157469

**Published:** 2023-10-25

**Authors:** Costina-Ruxandra Poetar, Nathan Bradley, Alexandra Voinescu

**Affiliations:** ^1^Department of Clinical Psychology and Psychotherapy, Babeș-Bolyai University, Cluj-Napoca, Romania; ^2^The International Institute for the Advanced Studies of Psychotherapy and Applied Mental Health, Babeș-Bolyai University, Cluj-Napoca, Romania; ^3^Department of Psychology, University of Bath, Bath, United Kingdom

**Keywords:** computer, mindfulness meditation, mood, simulator sickness, virtual reality

## Abstract

**Introduction:**

Mindfulness interventions are effective in improving mood, reducing stress, and increasing quality of life. New developments in technology bring important channels to deliver mindfulness interventions that can increase accessibility, such as the Internet, computerised interventions, mobile apps and recently, virtual reality (VR). The aim of the present study is to enhance our current understanding of the use of VR in mindfulness, namely we examined in a pilot randomised trial the efficacy of an immersive VR-based mindfulness approach compared to an active control (computerised-based mindfulness meditation) on improving mood. A secondary objective was to examine whether VR use resulted in simulator sickness which could affect user engagement.

**Methods:**

Forty-seven (*M_age_* = 29.22 *years*) healthy participants were randomly assigned to the experimental or control group.

**Results:**

A mixed 2X3 ANOVA showed a significant Time effect. Namely, negative emotions were reduced in both groups, with non-significant differences between groups. For positive emotions, on the other hand, our results showed no significant impact. Simulator sickness in VR was not present, according to *t*-test, making VR a safe delivery method.

**Discussion:**

Future research should investigate VR dosage and combine VR with other interventions (e.g., blended with face-to-face mindfulness interventions, with Internet-delivered interventions).

## 1. Introduction

Mindfulness has its roots in Buddhism and refers to the ‘ability to observe one’s physical sensations, emotions, and thinking having an open, nonjudgmental, and accepting attitude regarding one’s experiences’ (Kabat-Zinn, 2003). Several meta-analyses indicated that mindfulness-based programs (MBPs) are effective both in clinical (Goldberg et al., 2018) and in nonclinical populations (Khoury et al., 2015; Querstret et al., 2020) in improving mood and quality of life.

MBPs can be used for mental health promotion, as the results of a meta-analysis indicated a significant reduction in anxiety, depression, psychological distress and improved well-being when compared to no intervention (Galante et al., 2021). Recent developments in technology set the stage for different modalities to deliver MBPs, such as: desktop/computerised/web-based interventions, Internet-based interventions, mobile apps, or virtual reality (VR). Technology-enhanced MBPs are effective in reducing negative affect and promoting mindful awareness, with small to medium effect sizes for anxiety, depression, stress and mindful awareness (Victorson et al., 2020).

Different technology-enhanced MBPs have been investigated so far, with the most investigated forms of delivery being computerised/web-based, Internet-delivered or mobile apps for which evidence on their effectiveness/efficacy is coming from meta-analyses and systematic reviews. Small to moderate effects were reported for online MBPs on mental health outcomes (Spijkerman et al., 2016), for web-based MBPs (Sevilla-Llewellyn-Jones et al., 2018), and for mobile apps (Wu et al., 2022). However, a direct comparison of the many modalities through which mindfulness programs can be delivered has not been carried out. Rather, as previously stated, there is more research available on certain delivery methods than there is on equivalence, inferiority, or superiority of one delivery method over another.

Although several technology-enhanced MBPs have been investigated more than others, with numerous studies conducted on classic delivery methods (i.e. online/Internet based, desktop or mobile apps on screen-based devices, such as a smart tablet), several limitations exist, such as adherence to programme, motivation, number of sessions. These limitations with respect to existent screen-based interventions could be overcome by incorporating MBPs into immersive VR. Several benefits in comparison to standard, screen-based MBP, are related to the feelings of presence, decreased distraction from external stimuli as a result of the necessary attentional resources required, and greater motivation and adherence owing to its gamification features (Arpaia et al., 2022). In fact, in a study employing a mixed quantitative-qualitative method, participants mentioned that the VR setup restricted mind-wandering, facilitating their return to the environment (Seabrook et al., 2020). The same research indicated that elements such as selecting anchors are methods used to personalise the VR experience.

Even though research on the efficacy of VR-delivered MBPs is only beginning to emerge, the majority of it has been undertaken with clinical samples (e.g. generalised anxiety disorder, posttraumatic stress disorder; Navarro-Haro et al., 2019; Mistry et al., 2020), therefore, the efficacy of such promising interventions for non-clinical populations remains largely unstudied. Failla et al. (2022) conducted a systematic review on the mindfulness interventions mediated by VR systems in influencing mood and physiological status in non-clinical populations. The authors included seven studies in their qualitative synthesis and concluded that research on this topic is still in its early phases. VR mindfulness combined with neurofeedback study is superior to audio-guided meditation in a sample of healthcare workers in a hospital setting during the COVID-19 pandemic (Tarrant et al., 2022). In a student sample, VR mindfulness also reduced pre-exam anxiety more than the control condition (video-based meditation; Kaplan-Rakowski et al., 2021). On non-clinical participants preliminary research has been undertaken on the advantages of immersive VR-based MBPs delivery in improving mood. For instance, in a pilot study, Navarro-Haro et al. (2017) examined mood improvements while looking at the feasibility and acceptability of immersive VR for trained meditators. The results indicated that trained meditators were highly acceptive of the VR delivery method and that their mood improved with significantly lower sadness and greater relaxation ratings. Other preliminary evidence comes from a research that investigated the effectiveness of an immersive VR mobile app. Significant improvements in positive emotions and in state mindfulness were obtained, according to the findings (Seabrook et al., 2020). Furthermore, data coming from a randomised controlled trial, where VR-based MBP was compared to MBP and Relaxation designed to reduce stress in university students showed that participants in the VR-based MBP had the highest treatment adherence (Modrego-Alarcón et al., 2021).

Despite the existence of evidence-based interventions for distress, there is a significant gap in treatment access (Roberts et al., 2022). New modalities to deliver evidence based mental health interventions are urgently needed. The current study aimed to investigate the preliminary efficacy of an immersive VR-based MBP on improving mood in a nonclinical sample of participants, as compared to an active control, namely a screen-based MBP. As aforementioned, so far, no study compared the two conditions directly, but investigated their efficacy separately. Our main objective was to investigate if mood will be affected by MBP delivery mode and expected significant changes from baseline to post-meditation assessment and between VR and computer-based MBP. A secondary objective was to examine whether in the immersive VR condition participants will experience simulator sickness and the severity of this, and consequently, affect the effectiveness of this as a delivery method (Tian et al., 2022).

The following hypotheses were tested in line with the existing literature discussed above: (1) mood will significantly drop between baseline (T0) and post-stress test (T1) and increase post-meditation (T2). Due to the absence of prior direct comparisons between VR and computer-based MBP, we conducted an exploratory investigation to explore whether there will be significant differences in mood from post-stress test (T1) to post-meditation (T2) between VR-based MBP and computer-based MBP.

## 2. Materials and methods

### 2.1. Participants

Volunteer sampling was used to recruit participants. Study information was shared via social media and through the University of Bath Psychology Department Research Participation scheme.

Participants were provided with information and debrief sheets outlining their right to withdraw and data storage information. Participants had the opportunity to ask questions prior to starting the study and informed consent was gained. The information sheet provided advance warning that the study would include a stress test involving singing. To counteract distress caused by the stress test all participants completed a meditation. Information on support services was provided in the debrief. During the Short Sing-a-Song Stress Test (van der Mee et al., 2020) participants were told their performance will be recorded and investigated by musical professionals. The debrief rectified this misrepresentation.

Forty-nine participants partook in the study, but two cases were excluded as they completed the questionnaire responses for post-meditation without completing their designated condition. Of these 47 participants, 49% had previous VR experience, 43% reported playing video games a few hours a week or more and 39% of them had corrected vision. Furthermore, one participant failed to report their sex and age, so of the remaining 46 participants, most were women (59%) ranging in age from 19 to 70 (*M* = 29.22, *SD* = 12.71). Inclusion criteria were: age above 18, fluent in English, have normal or corrected vision or hearing, able to complete assessments alone. Exclusion criteria for participants were as follows: neurological conditions such as epilepsy, a diagnosis of depression in the past 12 months, severe motion sickness and/or any heart disorders.

### 2.2. Interventions

Two types of delivery methods of MBP interventions were investigated: a VR-based and a computer-based MBP. Interventions were comparable/similar in terms of duration (8 min), the single session format, and the stimuli presented.

#### 2.2.1. Virtual reality-based mindfulness-based program

**Figure 1:**
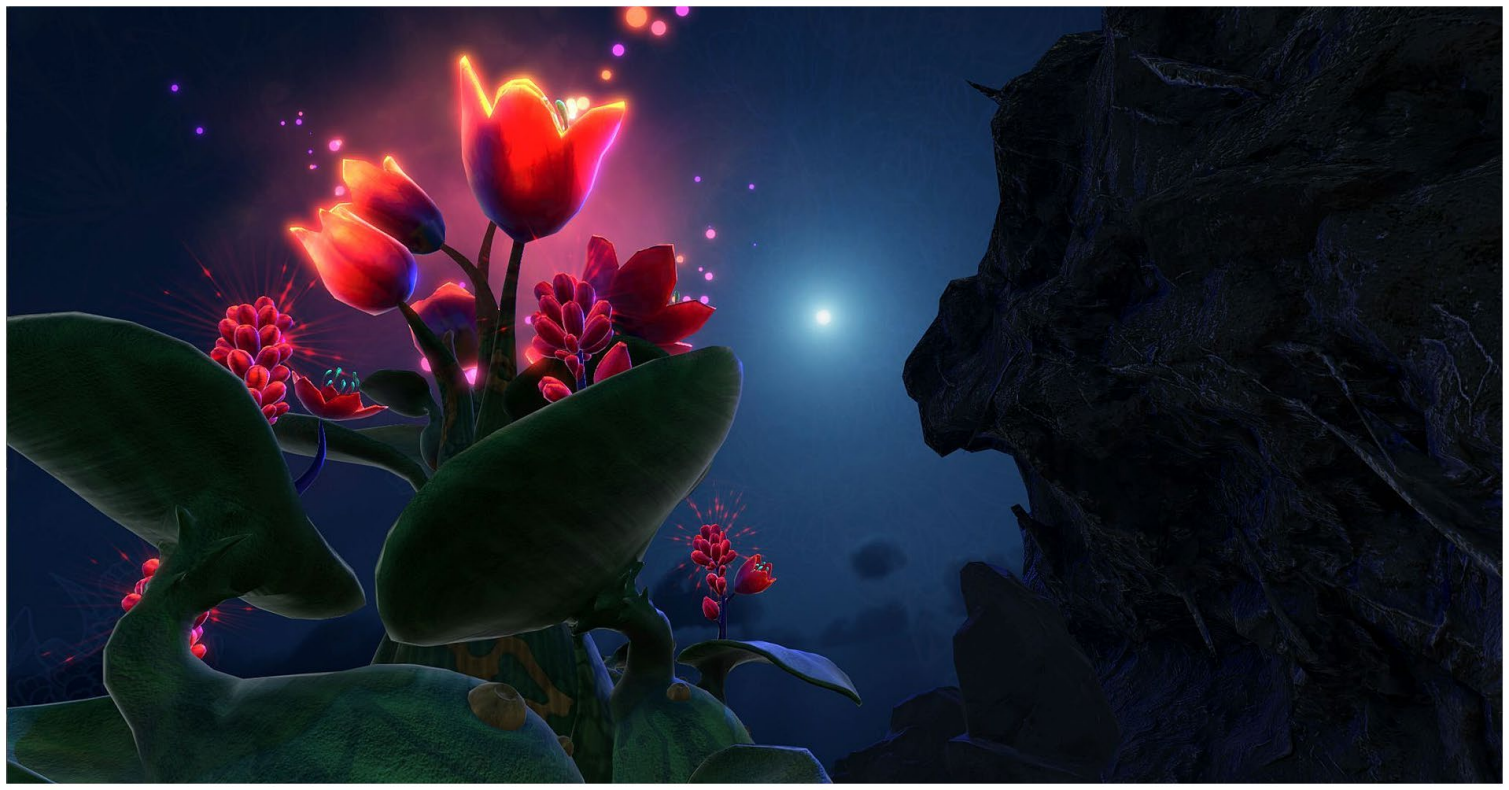
An image of the virtual environment during the virtual reality (VR) condition task.

An Oculus Go VR head mounted display was used with the following specifications: fast-switching 5.5″ diagonal LCD display with a resolution of 1,280 × 1,440 pixels per eye (2,560 × 1,440 combined), a refresh rate of 60–72 Hz and a field of view of 101 degrees. Audio was supplied with Behringer headphones and volume was set at a comfortable level for the participants which they could adjust themselves. Once the session, consisting of a mindfulness meditation (MM) free demo using the TRIPP application (Tripp, Inc 2018),^1^ started, users were able to look in any direction at different parts of the virtual environments. This application was recently tested (Holley et al., 2023) in a qualitative study with participants recruited from a residential substance use treatment centre. The meditation consisted of an auditory guided meditation, calm background music and a visual journey through a surreal virtual environment. Instructions were to help increase participation and awareness in the present moment with a focus on breathing. Any user input required was achieved by moving one’s head, such as for a minigame consisting of directing a bird by turning your head to capture coins and avoid obstacles. Within the session users ‘move’ to several different environments, see Figure 1, such as a large flower in a lake. The guided meditation session includes many of the common techniques and goals of traditional mindfulness meditation, with a particular emphasis on regulated breathing and directing attention to present somatic awareness. Total duration of the VR MPB was 8 min.

#### 2.2.2. Computer-based mindfulness-based program condition

The computer condition consisted of listening to an eight-minute audio-only guided MM video (Take Care Coaching, 2018) on a computer monitor. The guided meditation was accompanied by a calm music background and a picture of a woman meditating was displayed on the screen. Participants were instructed to focus on how they feel right now, in the present moment, to move their awareness to their breath. Key focus points were controlled breathing and current somatic awareness. Audio was again supplied with the same Behringer headphones at a comfortable volume that the participants could self-adjust. Total duration of the computer-based MPB was 8 min.

### 2.3. Instruments

#### 2.3.1. Primary outcomes

Negative and positive emotions. The Positive and Negative Affect Schedule (PANAS; Watson et al., 1988) consists of 20 items across two subscales. Ten self-rated items measure positive affect (PA) and 10 measure negative affect (NA) on a 5-point Likert scale from 1 (*Very slightly or not at all*) to 5 (*Extremely*). PA refers to an individual’s attentiveness and enthusiasm with items such as ‘Alert’ and ‘Inspired’ while NA refers to distress and negative emotions with items such as ‘Distressed’ and ‘Hostile’. The PANAS is scored by adding the PA and NA items, with a higher score indicating greater PA and NA. The PANAS has been found to have strong psychometrics with high reliability (Cronbach’s α = 0.89 for the PA scale and 0.85 for the NA scale respectively) in a non-clinical sample (Crawford and Henry, 2004). When examined under the current study, high Cronbach alpha values were also present for the entire PANAS scale at T0, T1and T2 with values of 0.83, 0.77 and 0.81, respectively.

#### 2.3.2. Secondary outcomes

Simulator sickness. The Virtual Reality Sickness Questionnaire (VRSQ; Kim et al., 2018) was included in order to assess whether simulator sickness was induced due to the VR condition. The scale consists of nine self-report items that measure simulator sickness in a virtual environment. The measure is comprised of two components with the first four items, such as ‘eyestrain’, relating to the oculomotor component and the last five items, such as ‘headache’, relating to the disorientation component. All items are measured on a 4-point Likert scale from 0 (*None*) to 3 (*Severe*). Scoring for the VRSQ is calculated by converting the component scores to percentages and then averaging the two component scores together. The VRSQ has been found to be highly sensitive, reliable (Cronbach’s *α* = 0.93) and valid in its measured constructs (Sevinc and Berkman, 2020). Reliability was also investigated under the current study at T1 and T2 and Cronbach alpha values of 0.79 and 0.87, respectively.

### 2.4. Stress test

A brief, seven-minute Sing-a-Song Stress Test (van der Mee et al., 2020) was employed to create a state of stress-induced low mood which was measured immediately after this task. The test was displayed on a computer monitor using Microsoft PowerPoint and setting the presentation to autoplay. All text was centred and set in Calibri font size 40. The test consisted of three reading conditions, a speaking condition and a singing condition. The first two reading conditions consisted of neutral messages about vacuum cleaners while the third reading condition instructed participants to say the word ‘vacuum’ twice when the countdown reached 0. Following on was another neutral message before an instruction to sing a song at the end of the next countdown which would be recorded and examined by musical professionals.

### 2.5. Procedure

**Figure 2:**
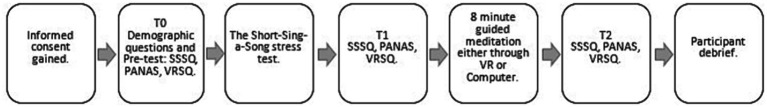
A flow chart of the study procedure.

This research was approved and complies with the ethical guidelines put forth by the University of Bath removed for blind review’s Psychology Research Ethics Committee (PREC; Ethics code: 21–145) as well as the British Psychological Society ethical standards and principles guidelines. Study design: a pilot randomised controlled trial was conducted with two groups regarding the meditation condition (VR, computer) assessed at three time points (baseline [T0], post-stress test [T1] and post-meditation [T2]). Data collection took place in-person in the University laboratory during COVID-19 related research restrictions between June–July 2021 but followed Governmental and university guidelines. All data was collected by two MSc Applied Clinical Psychology students as part of their dissertation projects. This was a one-time meditation session. After giving consent, participants started filling out demographic information and the first round of questionnaire responses through Qualtrics on a laptop (see Figure 2 for the procedure). They then completed the short Sing-a-Song Stress Test before completing the second round. Participants were randomly allocated to either the VR (*N* = 21) or computer condition (*N* = 26) using the randomise between function on Excel. Then, depending on their condition allocation, participants completed the VR or computer condition. The computer condition used the MBP video on the computer monitor and the VR used the TRIPP application on the Oculus Go. Due to COVID-19, the researcher was unable to set up the VR headset for the participants due to risk of contamination and therefore the participants set up the application themselves under the direction of the researcher who was in the same room, but maintained recommended distance between themselves and participants. Finally, the third round of questionnaire responses was completed on Qualtrics. The duration of the experiment was approximately 30–40 min.

Once the experiment had concluded, participants were reminded of their right to withdraw their data up until the end of data collection at a determined date. Participants were also shown a debrief form, informed of the deception regarding the recording of their singing and asked if they had any questions. Upon completion of data collection, two of the participants were randomly awarded a £50 voucher by feeding all the participant numbers through a computer randomiser.

Participants were randomly allocated to either the VR (*N* = 21) or computer condition (*N* = 26) using the randomise between function on Excel on a first come first served basis; however, the list was generated before participants’ enrolment by one of the researchers. Allocation concealment was not blinded because the researchers had access to the list of participants and their enrolment as they were the one to administer the procedure. There were no baseline differences between intervention groups (see Table 1) to suggest a problem with the randomisation process. As in other VR-based studies, blinding of participants and personnel was not possible (Voinescu et al., 2021); however, participants were blinded to study aim. Because this was a one-session procedure and in line with our University regulations in response to Covid-19 in order to minimise the spread of infections we were not allowed to use two experimenters for one session to interact with the participant and this is why the same experimenter administered the tasks and performed all the assessments. We tried to eliminate potential bias by administering the tasks on the computer and using well-validated scales.

**Table 1:** Participants demographics.

Variable	Summary statistics: Mean, SD, %		Independent *t*-test, *χ^2^*
	VR-based MBP (*N* = 21)	Computerised-based MBP (*N* = 26)	
Age	26.30 (10.94)	31.23 (13.56)	−1.32, *p* = 0.19
Gender			
Male	47%	53%	0.20, *p* = 0.66
Female	41%	59%	
Previous VR experience			
Yes	52%	48%	1.02, *p* = 0.31
No	38%	62%	
Gaming experience			
Yes	41%	59%	0.40, *p* = 0.53
No	50%	50%	
Education status			
Undergraduate	33%	67%	0.01, *p* = 0.93
Postgraduate	35%	65%	

### 2.6. Statistical analyses

Data was analysed in SPSS version 21. First, Shapiro–Wilk tests were conducted to examine the assumption of normality and this was violated by several variables. Levene’s test was conducted for assumptions checks of equal variance. Second, we conducted t-tests in order to compare the two groups across the variables of interest to establish equivalence in baseline. A mixed 2*3 Analysis of variance (ANOVA) was conducted with the delivery method as the between groups factor (computer, VR) and time as the within factor (T0, T1, T2). Effect sizes were reported using partial eta squared (*η_p_^2^*) and interpreted it as follows: *η_p_^2^* < 0.04 represents a small effect size, 0.04 < *η_p_^2^* < 0.25 represents a moderate effect size and *η_p_^2^* > 0.25 means a large effect size (Ferguson, 2009). To correct for multiple comparisons we used Bonferroni corrections for the significance level.

## 3. Results

### 3.1. Data handling

Initial data checks were conducted to ensure statistical analysis assumptions were met. As seen in Table 2, Shapiro–Wilk tests were conducted to examine the assumption of normality and this was violated by several variables, as supported by visual inspection of their normal Q-Q plots. Furthermore, inspection of skewness and kurtosis coefficient values for most variables were outside an acceptable range (Gravetter and Wallnau, 2014) of ±2.

However, analysis of variance (ANOVAs) remains a robust statistical test for data that violates distribution normality (Blanca et al., 2017). Furthermore, outliers were kept as some were of practical interest, such as a VR case with a high VRSQ (60) and a high PANAS NA score (22) at T2; highlighting in this case that simulator sickness may explain why negative emotions remained high. Additional checks on assumptions of equal variance using Levene’s test for the main analyses were non-significant.

### 3.2. Main analysis

To investigate our main objective results from a mixed 2 × 3 ANOVA showed that PANAS NA scores revealed a significant large main effect of time [*F*(1, 45) = 22.34, *p* < 0.001, *η_p_^2^* = 0.504], and non-significant main effects of meditation condition [*F*(1, 45) = 1.09, *p* = 0.300, *η_p_^2^* = 0.024] and response time*meditation condition interaction [*F*(1, 45) = 0.560, *p* = 0.575, *η_p_^2^* = 0.025]. Pairwise comparisons indicated a significant change from T0 to T2 (*p* = 0.002) and from T1 to T2 (*p* < 0.001). Our manipulation check indicated that our stress test failed to significantly increase negative emotions.

**Figure 3:**
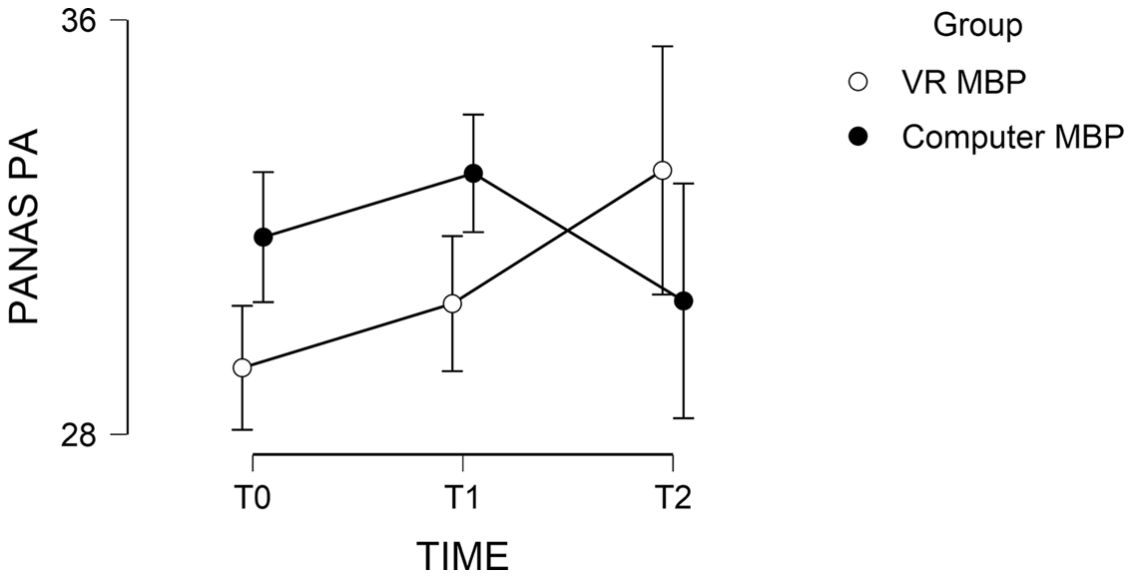
The mean PANAS PA score results for participants in the VR and computer conditions with standard error bars.

Analysis of the PANAS PA scores revealed non-significant main effects for time [*F*(1, 45) = 1.60, *p* = 0.207, *η_p_^2^* = 0.034] and meditation condition [*F*(1, 45) = 0.176, *p* = 0.677, *η_p_^2^* = 0.004] and a significant response time*meditation condition interaction [*F*(1, 45) = 6.37, *p* = 0.003, *η_p_^2^* = 0.025]. Simple effects analyses revealed that PANAS PA scores of participants in the VR and computer meditation conditions did not differ at T1, *t*(44) = 0.563, *p* = 0.576, or at T2, *t*(44) = 0.974, *p* = 0.335. Nevertheless, upon visual inspection of Figure 3, a cross-over interaction can be seen where PANAS PA scores increase in the VR condition group from T1 to while the PANAS PA scores decrease in the computer meditation group between T1 and T2.

A secondary objective was to assess whether VRSQ scores changed after being exposed to the VR. We conducted a paired sample *t*-test and results revealed that, on average, participants’ VRSQ scores in the VR-MBP group were not different between T1 and T2, *t*(20) = 0.938, *p* = 0.360. As a result, we decided against conducting a post-hoc Analysis of Covariance, examining whether significant differences would be detected between T1 and T2 for PA and NA scores when controlling for VRSQ scores at T2 (Table 2).

**Table 2:** Descriptive statistics and checks for all dependent variables for each meditation delivery condition.

Dependent variable	M (*SD*)		Skewness			Kurtosis	Shapiro–Wilk	
	VR	Computer	VR	Computer	VR	Computer	VR	Computer
PANAS PA-T0	30.52 (8.05)	32.84 (6.38)	−0.55	−0.61	−0.48	0.67	0.94	0.96
PANAS PA-T1	30.52 (8.16)	31.76 (6.73)	−0.19	−0.41	−0.18	−0.22	0.96	0.96
PANAS PA-T2	33.10 (10.00)	30.48 (8.21)	−0.57	−0.07	−0.69	−0.50	0.93	0.97
PANAS NA-T0	13.10 (2.91)	14.88 (6.27)	0.92	1.61	0.36	1.76	0.89*	0.77***
PANAS NA-T1	15.24 (5.22)	15.88 (5.97)	1.05	0.86	0.15	−0.06	0.85**	0.88**
PANAS NA-T2	11.29 (3.04)	11.40 (3.10)	2.78	2.48	7.91	5.69	0.51***	0.53***
VRSQ-T1	10.87 (12.87)	14.97 (14.97)	1.99	2.13	4.41	6.77	0.78***	0.80***
VRSQ-T2	7.50 (13.33)	10.17 (15.43)	3.45	2.01	13.03	4.79	0.53***	0.71***

*p < 0.05. **p < 0.01. ***p < 0.001. T0 = baseline. T1 = post-stress test. T2 = post-meditation.

## 4. Discussion

The current study aimed to investigate the efficacy of a one session VR MBP intervention as compared to an active control condition (computer MBP) in improving mood. Our findings indicated that VR MBP was as effective as computer MBP in improving mood. Specifically, as expected, a significant reduction in negative emotions was observed between T1 and T2, demonstrating that MBP was effective in reducing negative emotions independent of the delivery manner. The large effect size demonstrates the clinical implications of the present study, reinforcing the use of MBP as an intervention to improve mood using technological devices.

Contrary to our predictions, given that there were no changes in positive emotions, our results suggest that improvements in mood due to MBP may be caused by a reduction in negative emotions rather than an increase in positive ones. MBP allows the user to self-regulate their emotions to bring about a state of calm (Pepping et al., 2016). Therefore, mindfulness meditation may reduce negative emotions but will not affect positive ones as described and measured by the PANAS scale. Items used in the scale such as ‘Proud’ and ‘Strong’ would unlikely be affected by a MBP session that is focused on respiratory control and present moment somatic awareness. This is a novel finding within the meditation literature for a healthy sample as most studies either examine mood as a single construct (Zeidan et al., 2010) or analyse negative emotions exclusively (Feldman et al., 2010).

In addition, a cross-over interaction was found where positive emotions increased between T1 and T2 for the VR condition but decreased for the computer condition. This is in line with other findings which showed that the delivery of education materials through VR improved positive emotions whereas delivery through a computer decreased them (Allcoat and von Mühlenen, 2018). Our results regarding the non-significant Time effect for positive emotions are similar to those obtained by Bellosta-Batalla et al. (2020). In their study, the authors compared a single session mindfulness intervention with a control condition consisting of an emotion recognition exercise in a sample of university students. Findings indicated no changes in positive emotions, while negative affect and state anxiety decreased in the MBP group. It is possible that more sessions of MBP are needed in order to increase positive affect (e.g. 4 weeks or 8 weeks in traditional MBP; Demarzo et al., 2017). This idea is also supported by a study investigating the efficacy of an online MBP, where practice frequency was positively associated with positive affect (Bossi et al., 2022).

For the current VR setup and MBP application we found that a significant rise in simulator sickness symptoms did not occur, providing confidence in the use of VR delivery for MBP. This is important to emphasise as the efficacy of the method is irrelevant if its side effects deter user engagement. However, this finding is only relevant to similar VR setups and applications to the ones used in the current study. Different VR applications have been found to induce various amounts of simulator sickness (Munafo et al., 2017) and the VR headset technical specifications such as field of view (Adhanom et al., 2020) and latency (Stauffert et al., 2018) have been found to affect it too. To our knowledge, this examination of simulator sickness in users undertaking VR guided MBP is unique within the literature. Therefore, while informative for practitioners and developers when helping healthy users, further bespoke research should be conducted to examine potential side effects for other VR setups and applications.

Our study has important practical implications. Given the fact that VR technologies have become widespread and affordable, more people can access mindfulness training, therefore this method of delivery can help overcome accessibility barriers. As a result, a wider spectrum of people can benefit from VR-based MBP at their own comfort requiring limited resources. There is evidence that mindfulness interventions can promote mental health in non-clinical samples (Galante et al., 2023), with a reduction in psychological distress up to 6 months post-intervention. Initial qualitative evaluation of a VR-based MBP with both patients and staff indicated that this intervention could be feasible to use with clinical samples, specifically with people with substance use disorders (Holley et al., 2023). Moreover, it seems that delivering mindfulness interventions through VR can help overcome resistance or difficulties encountered when practicing traditional mindfulness (e.g. by decreasing distractions, both external and internal mind-wandering; Holley et al., 2023). Combining VR with traditional mindfulness practice can result in increased treatment adherence, as indicated by an RCT that compared MBP with MBP plus VR and relaxation alone (Modrego-Alarcón et al., 2021). Results of this study showed that while both conditions involving MBP had superior results on stress as compared to the relaxation group, retention rates and session attendance were higher for the combined condition (MBP + VR).

Several limitations of the present study must be considered before interpreting the results. A first limitation is related to the small sample size, which even though was estimated based on recommendations for conducting pilot studies (Whitehead et al., 2016), may still be underpowered to detect small differences between the two active conditions. Another limitation is related to the uneven distribution of participants across conditions (21 participants in the VR-based MBP vs. 26 in the computer-based MBP). This imbalance arose as we could not enrol more participants due to data collection limitations because of the COVID-19 pandemic and project time constraints, therefore the randomisation sequence generated in the Excel spreadsheet could not be filled. Future RCTs, with larger sample size are needed. For a small effect size, and power of 80%, three measurements and a correlation of 0.5 between measurements, a minimum 236 participants would be needed according to Gpower (Faul et al., 2007). For our study, we computed *post-hoc* achieved power with GPower. Results showed that for NA for the response time*meditation interaction effect power was 65%. A third limitation pertains to the fact that we did not measure participants’ previous mindfulness training. This could have influenced participants’ understanding of mindfulness concepts, ultimately leading to a more effective practice of the interventions.

A fourth drawback is related to the use of the stress induction task, given that it did not lower mood as it was already generally high. As all our data was collected through self-reported instruments, future studies should employ clinician ratings of relevant outcomes. Blind clinician assessors could rate participant’s psychological distress. The study was not pre-registered; however, it has undergone ethical approval by the Department of Psychology’s ethical committee where details concerning study’s aim, objective, hypothesis, inclusion/exclusion criteria, sample size were provided. Another aspect that needs to be considered is related to the duration of the intervention. Even though previous meta-analyses indicated that single-session mindfulness interventions are effective in improving negative mood, it seems that more sessions are needed in order to have a more stable effect in time (Schumer et al., 2018). Furthermore, in the literature on VR mindfulness interventions there is a high heterogeneity regarding VR single-sessions mindfulness interventions, with duration between 5 and 30 min according to a recent systematic review (Ma et al., 2023). As far as we know, there are no studies comparing single-session VR mindfulness with multiple VR mindfulness sessions, therefore, further research is needed to establish the optimal dosage of VR sessions. Finally, future research should inform us on the optimal dosage of VR MBP through factorial designs, and about its long-term efficacy given that a single session of VR MBP shows promising results on improving mood.

## 5. Conclusion

The main objective of our study was to compare two delivery methods in MBP, namely a VR-based MBP with a computerised MBP delivered via a single session. Our results indicated that while negative affect improved in both conditions, positive mood increased only in the VR-based MBP. In conclusion, a VR-based MBP could be a more promising alternative to classical meditation. This VR setup and guided MM session did not cause simulator sickness, which suggests that VR-based meditation could work on a larger scale. This study has important practical implications given the increased accessibility of VR technologies which could reach a wider audience and promote mental health.

## Data availability statement

The raw data supporting the conclusions of this article will be made available by the authors, without undue reservation.

## Ethics statement

The studies involving humans were approved by Psychology Ethics Committee – University of Bath. The studies were conducted in accordance with the local legislation and institutional requirements. The participants provided their written informed consent to participate in this study.

## Author contributions

C-RP contributed to the formal analysis, data curation, writing – review and manuscript editing. NB collected the data and wrote the first draft of the manuscript. AV contributed to the conception and methodology of the study. All authors contributed to the article and approved the submitted version.

## Footnotes

^1^https://www.tripp.com/
